# Dermatology

**Published:** 1894-01

**Authors:** 


### DERMATOLOGY.

---

Dr. L. A. Duhring read a paper on dermatology, at the last meeting of the American Medical Association, in which he urged close attention to the constitutional condition in dermatological cases.

Dermatology, of course, means the "science of the skin," literally. Aside from the exanthemata, dermatology embraces all eruptions, alterations, anomalies or growths of the skin. Some are purely local and need only local treatment, be it medicinal or mechanical. Others depend, directly or indirectly, upon a general condition. Few are constitutional. Syphilis is. Many have the depressing effect of a constitutional or internal disease as a predisposing condition, but are not symptomatic of this condition. You must recognize and treat the general disorder, but the cure of the skin disease will be hastened by proper local treatment; sometimes all will be well but the skin trouble, which will finally yield only to local measures. It is the physician's duty to relieve all defects of the general health, if he can.

---

MARRIED.—Dr. Luther B. Grandy and Miss Hattie Smart were married at the residence of the bride's parents, 263 Peachtree street, on Thursday, December the 14th, at 3 P. M. They left for a bridal tour at 6 P. M. They will be "at home" after January 1st. Dr. Grandy is the Managing Editor of THE JOURNAL. The rest of THE JOURNAL extend heartiest congratulations.

---

ELECTION OF OFFICERS ATLANTA SOCIETY OF MEDICINE.—The following officers have been elected by the Atlanta Society of Medicine for the year 1894: President, Dr. J. W. Duncan; Vice-President, Dr. C. D. Hurt; Secretary, Dr. R. R. Kime; Treasurer, Dr. L. B. Grandy; Corresponding Secretary and Librarian, Dr. N. O. Harris.

---

Dr. Ross P. Cox, of Rome, was in the city a few hours the other day, for the purpose of attending a meeting of the State Medical Association Committee on Programme.

